# MiR-27a-3p Promotes Non-Small Cell Lung Cancer Through SLC7A11-Mediated-Ferroptosis

**DOI:** 10.3389/fonc.2021.759346

**Published:** 2021-10-13

**Authors:** Xuan Lu, Ningning Kang, Xinxin Ling, Ming Pan, Wenjing Du, Shan Gao

**Affiliations:** ^1^ Department of Pharmacology, Basic Medical College, Anhui Medical University, Hefei, China; ^2^ The First Affiliated Hospital, Anhui Medical University, Hefei, China; ^3^ Department of Integrative Medicine, Huashan Hospital, Fudan University, Shanghai, China

**Keywords:** miR-27a-3p, SLC7A11, ferroptosis, non-small cell lung cancer, MDA progress

## Abstract

**Background:**

Ferroptosis is a newly generated regulatory cell death promoted by the accumulated lipid-based reactive oxygen species (ROS). Solute carrier family 7 member 11 (SLC7A11), the cystine/glutamate antiporter, is known as a ferroptosis executor that exhibits a positive correlation with carcinoma progression because of antioxidant function. Nonetheless, it is yet unclear on the understanding of ferroptosis regulation in lung cancer.

**Methods:**

Database, qRT-PCR, Western-blot (WB), and immunohistochemistry were utilized to determine SLC7A11 expression and function, as well as gene iron related to necrosis in clinical tissue specimens and cells; a ferroptosis inducer, inhibitors, and SLC7A11 lentivirus were used to confirm SLC7A11’s biological activity in cell viability, oxidative stress, lipid peroxidation, and iron ion enrichment in non-small cell lung cancer (NSCLC) in different cells; lentivirus was used to infect lung adenocarcinoma cell lines to acquire miR-27a-3p overexpression and knockdown cell lines, and to detect SLC7A11 level through qRT-PCR and WB. The influence of upregulated/downregulated miR-27a-3p on ferroptosis and other related biological characteristics of lung adenocarcinoma cell lines was detected.

**Results:**

Upregulated SLC7A11 was shown in NSCLC patients and cells, and increased SLC7A11 had a relation to the poorly prognostic status of NSCLC patients. Besides, a novel miRNA, miR-27a-3p, was an essential modulator of ferroptosis *via* directly targeting SLC7A11 in NSCLC cells. Overexpressing miR-27a-3p led to SLC7A11 suppression *via* directly binding to its 3’-UTR, followed by the reduction of erastin-caused ferroptosis. In contrast, inhibited miR-27a-3p resulted in an increase in NSCLC cells’ sensitivity to erastin. Of importance, the accumulated lipid ROS and cell death of iron peptide mediated by anti-miR-27a-3p can be eliminated by impeding the glutamylation process. Our literature collectively uncovered that miR-27a-3p modulated ferroptosis by targeting SLC7A11 in NSCLC cells, illustrating the important role of miRNA in ferroptosis.

**Conclusion:**

MiR-27a-3p modulates ferroptosis *via* targeting SLC7A11 in NSCLC cells, implying the significant role of miR-27a-3p/SLC7A11 in ferroptosis.

## Introduction

It was reported that lung cancer (LC) was still the leading inducer of carcinoma-associated mortality around the world. The widely occurred type of LC was non-small cell lung cancer (NSCLC), accounting for 85% of all the LC cases. The survival time of LC after diagnosis is 3 months to 1 year, decided by stages I–IV (1). Advances in treating LC, consisting of better surgical resection, radiotherapy, and chemotherapy, along with molecular and targeted therapies, performed well in improving LC patients’ survival rate (2). Nonetheless, early detection, strong therapeutic options for controlling neoplasm development, and methods for predicting the survival status and/or the response after treatment were the present challenges with regard to clinicians. Patients with LC often experienced the recurrence and metastasis of neoplasm, contributing to disappointing overall survival (OS) (3). The results of the multivariate analysis revealed that gender, age, TNM comprehensive stage, and maximum tumor diameter were independent factors affecting the prognosis of patients with NSCLC after radical resection (4). The younger the age of diagnosis, the earlier the TNM comprehensive stage, and the smaller the maximum diameter of the tumor, the better the prognosis of the patients. Therefore, a preferable understanding of the pathogenesis, molecular alterations, and new therapies of LC is helpful to detect LC at an early stage, explore efficacious treatment strategies against neoplasm pathology and development, and forecast therapeutic response and patients’ survival.

Cell death functions importantly in many situations, i.e., holding homeostasis in disease development and prevention (5). In addition to apoptosis, a new programmable form of cell death, ferroptosis, has also recently attracted people’s attention. Ferroptosis is presently recognized as regulated cell death, with the characteristics of the accumulated lipid peroxidation products and deadly reactive oxygen species (ROS) from iron metabolism, and is thought to be different from other kinds of cell death in genetics, biochemistry, and morphology (6, 7). It is characterized by glutathione peroxidase 4 (GPX4) inactivation, which causes the accumulation of iron-dependent lipid peroxides in cells, leading to cell death (8, 9). Another key executor involved in the ferroptosis pathway is the cystine/glutamate transporter (SLC7A11, SLC3A2), maintaining the uptake of cystine and the excretion of glutamate. The depletion of glutathione activates ferroptosis (10, 11). The SLC7A11 functions to import cystine for glutathione biosynthesis and antioxidant defense and is overexpressed in multiple human cancers. As a critical modulator of intracellular redox balance, targeting SLC7A11 is considered a promising therapeutic opportunity for cancer treatment. Growing researches has shown that ferroptosis is related to several human diseases, comprising neurodegenerative diseases, ischemic reperfusion injuries, and renal degeneration (12). Additionally, many carcinoma cells are sensitive to ferroptosis inducers, while little is known about LC. What is more, erastin, a ferroptosis inducer, can ameliorate the effectiveness of chemotherapy drugs, such as temozolomide, cisplatin, cytarabine, and adriamycin (13). Herein, to induce and enhance ferroptosis was a prospective carcinoma tactic. Nevertheless, it seems to be drug or cell type-specific regarding these molecules players’ role in ferroptosis. Therefore, ferroptosis’s regulatory mechanism in the development of LC is yet unclear.

MicroRNAs (miRNAs or miRs) with a length of approximately 25 nt, belonging to small noncoding RNAs, icroRNAs (miRNAs or miRs), modulate gene expression *via* binding to the 3’UTR of target mRNA. A given miRNA can simultaneously inhibit the expression of multiple target genes based on sequence homology and thereby has significant influence on gene networks and cellular signaling pathways (14). Tianzhi Huang et al. found that microRNA-93 regulates tumorigenicity and therapy response of glioblastoma by targeting the expression and/or activity of key autophagy regulators (15). Therefore, miRNA exhibits importance in regulating protein expression after translation. As previously described, miRNAs participated in the occurrence, progression, and control of LC (16). To date, there is not yet any microRNA that directly regulates ferroptosis in lung cancer. In our context, we attempt to clarify the role of SLC7A11 in NSCLC and unearth the hidden mechanism. Our data indicated that SLC7A11 was dramatically raised in NSCLC patients and cell lines, implying that it was an indicator of poor prognostic status. We, for the first time, demonstrate that ferroptosis mediates the regulation of NSCLC development *via* targeting the miR-27a-3p/SLC7A11 pathway, revealing that, therefore, miR-27a-3p/SLC7A11 was an encouraging treatment biomarker and target for patients with NSCLC.

## Materials and Methods

### Microarray Data

GSE27262, GSE102287, GSE116959, GSE118370, and GSE19945 were acquired from the Gene Expression Omnibus (https://www.ncbi.nlm.nih.gov/geo/) on the basis of the platform of the Affymetrix Human Genome U133 Plus 2.0 Array, Affymetrix Human Genome U133 Plus 2.0 Array, Agilent-039494 SurePrint G3 Human GE v2 8x60K Microarray 039381, Affymetrix Human Genome U133 Plus 2.0 Array, and Agilent Human 0.6K miRNA Microarray G4471A, respectively.

### DEG Identification

GEO2R (http://www.ncbi.nlm.nih.gov/geo/geo2r), as a free website toolset, was utilized to compare gene expression data of multiple groups and to identify the differentially expressed genes (DEGs) and differentially expressed miRNAs (DEMs) between the samples of neoplasm and normal control. A p-value <0.05 meant that there was a significant difference.

### KEGG Pathway Analysis

The Kyoto Encyclopedia of Genes and Genomes (KEGG) pathway analysis was applied to identify molecular interaction and relation networks through the Database for Annotation Visualization and Integrated Discovery (DAVID; http://david.ncifcrf.gov/). A p-value <0.05 meant that there was a significant statistical difference. The cutoff criteria were considered as a false discovery rate (FDR) <0.01 and gene count >2.

### Tissue Collection and Ethics Statement

A total of 90 NSCLC tissues and paired adjacent normal ones were taken from patients undergoing primary surgery at the Department of Thoracic Surgery in Huashan Hospital, Fudan University, between February 2016 and July 2020. Referring to the classification tumor node metastasis (TNM) and the standard of the World Health Organization (WHO), all tissue samples were staged and graded by an experienced pathologist. All samples that were immersed in RNA Later stabilization solution (Qiagen, Germany) were maintained in liquid nitrogen and preserved at -80°C. Patients were not subjected to any treatment against carcinoma prior to surgical resection. Our research was approved by the institutional ethics committee of Huashan Hospital of Fudan University, and the participants signed a written informed consent form.

### Cell Culture and Transfection

Human NSCLC cell lines were ordered from the National Collection of Authenticated Cell cultures, and Beas-2B cell lines were obtained from MingZhou bio company. NSCLC cell lines were maintained in DMEM (Gibco, USA) with 10% FBS (HyClone, USA) and 1% Penicillin/Streptomycin (Life Technologies, UK) under a 37°C incubator with 5% CO_2_. Beas-2B cells were maintained in LHC-9 medium with 0.5 ng/ml EGF, 500 ng/ml hydrocortisone.

A549 cells were transfected with shRNAs (sh-Scrambled and two shRNAs specifics for SLC7A11) and miRNAs (miRNA mimics and controls) by Lipofectamine 3000 (Invitrogen, USA) as manufacturers described. Puromycin (2 µg/ml, Sigma-Aldrich, USA) was added into cells for subsequent screening until stabilization. SLC7A11 cDNA was ligated into lentiviral construct (GenePharma) for subsequent studies.

### qRT-PCR

TRIzol (1 ml, Invitrogen) was employed to harvest the whole RNA, followed by reverse transcription with the use of a First Strand cDNA Synthesis Kit (Thermo Scientific, USA) as manufacturers instructed. qRT-PCR was taken to quantify the RNA level using a SYBR Premix ExTaq Reverse Transcription PCR kit (Takaka, China). GAPDH was utilized as a normalized control. All primers were listed in **Supplementary Table 1**.

### Cell Viability Assay

Cell Counting Kit (CCK-8) (Yesen, China) was employed to detect the cell viability in triplicate for 3 consecutive days at 450 nm by a microplate reader (Thermo Scientific, USA).

### Flow Cytometry

Fluorescence activated cell sorting (FACS) was employed to analyze cell proliferation at post-transfection. A total of 1×10^4^/well were incubated with Annexin V-FITC and propidium iodide (Yeasen, China) for 10 min at 4°C in the dark. Subsequently, the cells were rinsed with buffer two times and resuspended by 500 μl of buffer for flow cytometry analysis.

### Intracellular ROS Analysis

Dihydroethidium (DHE, Merck KGaA) was used to stain ROS, followed by flow cytometry detection as previously described (17). In short, cells were digested and risned and then mixed with 1.25 μM DHE for 30 min at 37°C in the dark. Fluorescence at 610 nm was measured on a FACS Calibur™.

### Iron Assay

The iron analysis kit (Abcam) was employed to measure the intracellular ferrous (Fe^2+^) level as manual depicted. Taken briefly, we collected samples, washed by precold PBS and homogenized in precold five volumes of iron analysis buffer. We harvested the supernatant and added an iron-reducing agent into every allocation, followed by incubation for 30 min. Each mixture was incubated with iron probes and incubated for 60 min. Subsequently, the OD (optical density) value of 593 nm was measured by a colorimetric microplate reader.

### Western Blot

Whole protein was harvested in samples by lysis buffer (Beyotime, China). Proteins (25 µg) with the corresponding volume of loading buffer were boiled in water bath for 10 min. SDS-PAGE gel was used to resolve the proteins and then transferred onto PVDF membranes (Millipore, USA). Primary and secondary antibodies were incubated as indicated. xCT/SLC7A11 (CST,12691), GPX4 (CST, 52455), and GAPDH (CST, 5174) were used in this study.

### Immunohistochemical

The rehydrated sections were retrieved by citrate buffer for 3 min at 100°C and incubated with primary antibodies at 4°C overnight. On the following day, second antibody (goat anti-rabbit IgG) was incubated with them at RT for 30 min. The washed sections were stained with diaminobenzidine and captured under a microscope. The images were analyzed by Image Pro Plus software (Media Cybernetics, USA).

### Luciferase Assay

We utilized TargetSan (http://www.targetscan.org/vert72), miRanda (http://www.microrna.org/microrna/home.do), microT (http://www.microrna.gr/microT), PITA (http://genie.weizmann.ac.il/pubs/mir07/mir07_data.html), miRmap (http://mirmap.ezlab.org), and PicTar (http://www.pictar.org/) databases to predict the upstream gene of SLC7A11. miR-27a-3p was chosen. The 3’UTR region with the miR-27a-3p binding site was cloned into pGL3-basic luciferase construct (Promega, USA). Cotransfection of luc-SLC7A11-wt/luc-SLC7A11-mut with miR-27a-3p mimic/NC mimic into A549 cells was performed by Lipofectamine 3000 (Invitrogen). At 48 h post-transfection, luciferase activity was measured by a Dual-Luciferase Reporter Assay System (Promega) as manufacturers instructed. Renilla luciferase was regarded as normalized control.

### Data Analysis

The represented data were shown as the mean ± SEM from three separate experiments in triplicate. Statistical significance was determined by the unpaired Student’s t test, *p < 0.05.

## Results

### SLC7A11 Is Obviously Upregulated in NSCLC Cell

**Figure 1:**
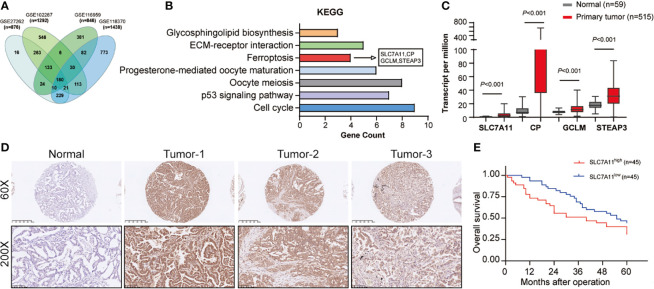
SLC7A11 expression in NSCLC tissues and its clinical significance. **(A)** Bioinformatic interrogation of Gene Expression Omnibus (GEO) datasets. **(B)** The cellular processes that were positively correlated with NSCLC according to KEGG analysis. **(C)** Expression level of SLC7A11, CP, GCLM, and STEAP3 in the TCGA-LAUD database. **(D)** The SLC7A11 protein levels in NSCLC patients were analyzed by immunohistochemistry. **(E)** Kaplan–Meier overall survival (OS) curves according to SLC7A11 expression levels.

First, we performed bioinformatic interrogation of GEO datasets. In total, 876, 1,292, 846, and 1,438 DEGs were obtained from GSE27262, GSE102287, GSE116959, and GSE118370, respectively. A total of 180 genes were screened out in all four datasets (**Figure 1A**). Furthermore, KEGG pathway analysis showed seven identified pathways including ferroptosis with four DEGs, SLC7A11, CP, GCLM, and STEAP3 (**Figure 1B**). To verify the expression trends of SLC7A11, CP, GCLM, and STEAP3 in NSCLC, TCGA-LAUD data were obtained and reanalyzed. We found that all the four DEGs were indeed upregulated in NSCLC (**Figure 1C**). Recent studies revealed that SLC7A11 overexpression promotes tumor growth partly through suppressing ferroptosis. As the upstream and key gene in the ferroptosis pathway, SLC7A11 was selected for further study.

A total of 90 NSCLC patients were classified into two groups in the light of the median value to assess in-depth the association of SLC7A11 expression with clinicopathological traits. First, IHC array was conducted to determine the SLC7A11 level in patients with NSCLC, and the result showed that its levels significantly increased in NSCLC patients’ tissues (**Figure 1D**). Our data revealed that SLC7A11 expression had an association with tumor size (chi-square test, *P* = 0.009), smoking history (chi-square test, *P =* 0.010), lymph node metastasis (chi-square test, *P* = 0.009), and TNM stage (chi-square test, *P* = 0.013) (**Table 1**). Nonetheless, no obvious correlation was observed between the SLC7A11 level and age, gender, differentiation, and primary location (chi-square test, *P* > 0.05; **Table 1**). Collectively, our results indicated that SLC7A11 may function essentially in the progression of NSCLC. In addition, the Kaplan–Meier method analysis revealed that the overall survival (OS) rate in the highly expressed SLC7A11 group was largely lower than that in the lowly expressed SLC7A11 group (**Figure 1E**).

**Table 1:** Multiple variables cox proportional hazards analysis in 90 NSCLC patients.

Correlation between SLC7A11 expression and clinicopathological parameters of NSCLC				
Clinicopathological parameters	N of cases (n=90)	Relative expression of SLC7A11		
		Low	High	P-value
Age (years)				0.753
<65	38	17	21	
>65	52	25	27	
Gender				0.887
male	50	23	27	
female	40	19	21	
Differentiation				0.337
well, moderate	25	12	13	
poor	65	24	41	
Tumor size				0.009
<4cm	41	34	7	
>4cm	49	28	21	
Primary location				0.846
left lung	42	20	20	
right lung	48	23	25	
Smoking history				0.010
ever or now	36	12	24	
never	54	33	21	
Lymph node metastasis				0.009
positive	47	21	26	
negative	43	31	12	
TMN stage				0.013
I	33	25	8	
ll/lll	57	28	29	

### SLC7A11 Induces Ferroptosis in NSCLC Cells

**Figure 2:**
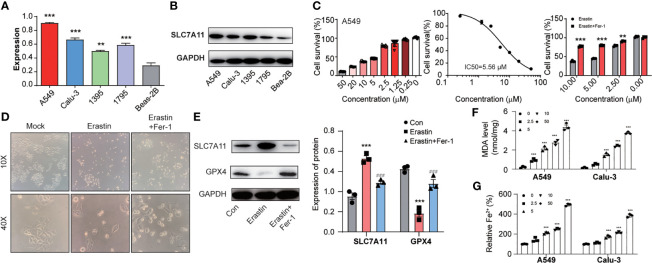
The lung cancer cells with high expression of SLC7A11 could induce ferroptosis. **(A)** and **(B)** SLC7A11 expression level in different NSCLC cells. **(C)** CCK8 assay was used to detect cell survival levels of A549 induced by erastin and erastin plus ferrostatin-1 at different concentrations. **(D)** Images analysis of A549 cells after erastin and erastin plus ferrostatin-1 treatment. **(E)** Expression levels of SLC7A11 and GPX-4 after erastin and erastin plus ferrostatin-1 treatment. A549 cells with treatment of multiple concentrations of erastin for 24 h were subjected to analysis. [There was significant difference between con and Erastin (***P < *0.001*). There was significant difference between Erastin and Erastin+Fer-1 (^###^ *P* < *0.001*)]. The lipid formation levels **(F)** and intracellular Fe^2+^ accumulation levels **(G)** on A549 and Calu-3 cells during the erastin induced ferroptosis progress. **P < 0.01 and ***P < 0.001.

Next, we identified SLC7A11 mRNA and protein expression in NSCLC cells. SLC7A11 was higher in NSCLC cells than in normal control (**Figures 2A, B**). Next, we analyzed erastin activity in A549 and Calu-3 cells. Both erastin (IC50 (A549) = 5.56 μM; IC50 (Calu-3) =2.72 μM) could induce cell death in A549 and Calu-3 cells, which could be reversed after treatment of ferrostatin-1 (ferroptosis inhibitor) (**Figure 2C** and **Supplementary Figure S1**). We used A549 cells for further analysis. Images showed erastin induced A549 cells around, swelled, and rescued by ferrostatin-1 (**Figure 2D**). GPX-4 is a ferroptosis maker, which protects cells against membrane lipid peroxidation. Notably, SLC7A11 expression increased and GPX-4 significantly attenuated during ferroptosis process (**Figure 2E**). Considering oxidative stress, lipid peroxidation, and iron accumulation were major signaling processes in generating ferroptosis, we focused on these three indicators. First, we found that erastin led to ROS accumulation in both A549 and Calu-3 cells and rescued by ferrostatin-1. Similarly, the H_2_O_2_ induced ROS could not be inhibited by ferrostatin-1, which confirmed that the oxidative stress happened during ferroptosis (**Supplememntary Figure S2**). Considering malondialdehyde (MDA) is one of primary lipid peroxidation end products, we investigated whether MDA was gathered in NSCLC cells. **Figure 2F** illustrated that the MDA level was raised by erastin with dosage increasing. Besides, ferrous iron (Fe^2+^) is another important factor leading to ferroptosis (**Figure 2G**). We also found the intracellular iron concentrations were increased depending on the ferroptosis progress.

### Silencing SLC7A11 Inhibits NSCLC Cell Proliferation and Lipid Peroxidation

**Figure 3:**
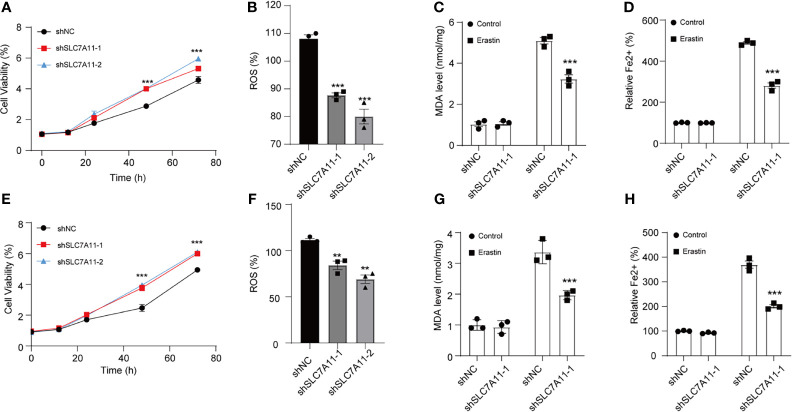
SLC7A11 modulates ferroptosis induced by erastin in NSCLC cells. **(A)** Determination of the viability in SLC7A11 knockdown A549 cells with erastin (5 µM) treatment for 24 h. The intercellular ROS **(B)**, MDA **(C)**, and Fe^2+^ levels **(D)** were significantly suppressed abrogated on SLC7A11 knockdown cells. **(E)** Cell viability of SLC7A11 knockdown Calu-3 cells with erastin (5 µM) treatment for 24 h. The intercellular ROS **(F)**, MDA **(G)**, and Fe^2+^ levels **(H)** were significantly suppressed abrogated on SLC7A11 knockdown cells. **P < 0.01 and ***P < 0.001.

Next, SLC7A11 was ablated n by SLC7A11 shRNAs in two NSCLC cell lines to investigate SLC7A11’s role (**Supplementary Figure S3**). Silencing SLC7A11 facilitated A549 cell viability compared with the control group (**Figure 3A**). Consistently, MDA accumulation assay demonstrated that the reduction of SLC7A11 resulted in the decrease in ROS and MDA accumulation under the stimulation of erastin in A549 cells (**Figures 3B, C**). Thus, we further uncovered the impacts of ablated SLC7A11 on Fe^2+^ levels. The intracellular Fe^2+^levels were reduced in A549 cells upon erastin treatment (**Figure 3D**). The same results were also illustrated in Calu-3 cells (**Figures 3E–H**). Collectively, our results suggested that SLC7A11 exhibited a key role in ferroptosis.

### miR-27a-3p Directly Targeted SLC7A11

**Figure 4:**
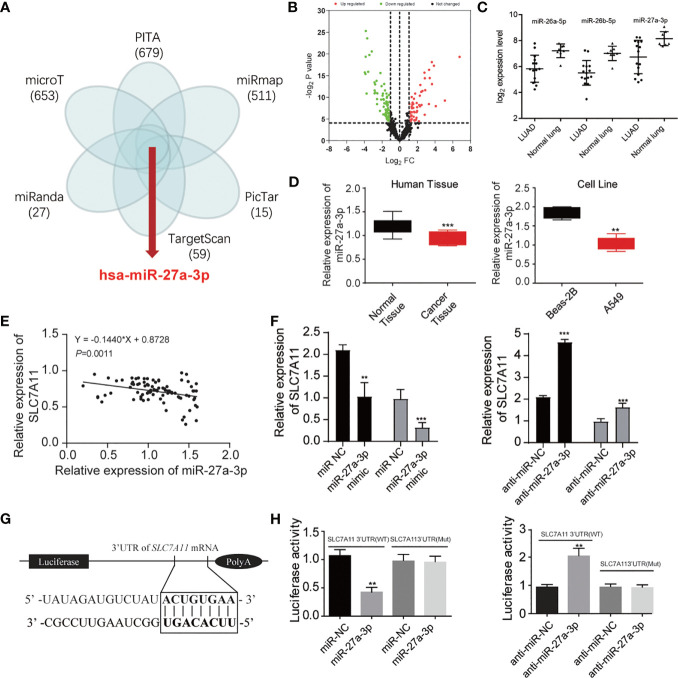
miR-27a-3p is the regulator of SLC7A11 protein. **(A)** Predicted the miRNAs targeting SLC7A11 using TargetSan, miRanda, microT, PITA, miRmap, and PicTar databases. **(B)** Volcano plot indicating the downregulated and upregulated microRNA in NSCLC tissues. **(C)** Three overlapping miRNAs were screened out including miR-26a-5p, miR-26b-5p, and miR-27a-3p. **(D)** Measurement of miR-27a-3p expression in the tissues and cell lines of NSCLC. **(E)** The correlation of SLC7A11 with miR-27a-3p. **(F)** Determination of the SLC7A11 expression level in miR-27a-3p mimic and anti-miR-27a-3p treated A549 cells, respectively. **(G)** Prediction of miR-27a-3p’s e binding sites in SLC7A11. **(H)** Detection of luciferase activity of A549 cells with cotransfection of SLC7A11 (WT) or SLC7A11 (MUT) and miR-27a-3p or anti-miR-27a-3p. **P < 0.01 and ***P < 0.001.

We explored in-depth the mechanism of SLC7A11 in ferroptosis. The miRNAs targeting SLC7A11 were predicted using TargetSan, miRanda, microT, PITA, miRmap, and PicTar (**Figure 4A**). A total of five overlapping miRNAs were screened out including miR-1297, miR-26a-5p, miR-26b-5p, miR-27a-3p, and miR-27b-3p. We further detected these miRNAs’ expression in the GSE19945 dataset, and the results showed that miR-26a-5p, miR-26b-5p, and miR-27a-3p were downregulated in NSCLC (**Figures 4B, C**). Among the three miRNAs, miR-27a-3p expression was remarkably decreased in cancer tissues and A549 cells (**Figure 4D**). The association of SLC7A11 mRNA expression with miR-27a-3p was further investigated. Pearson correlation analysis showed that SLC7A11 mRNA expression displayed a greatly negative correlation with miR-27a-3p (r = 0.797, *P* < 0.001) (**Figure 4E**). SLC7A11 mRNA expression was significantly upregulated, and ablating miR-27a-3p gave rise to increasing SLC7A11 mRNA level in miR-27a-3p mimic-transfected NSCLC cells (**Figure 4F**). Luciferase reporter assay was carried out to verify the relation of SLC7A11 with miR-27a-3p, and the data revealed that decreased luciferase activity was observed in A549 cells with cotransfection of miR-27a-3p and WT-SLC7A11-3’UTR rather than the control group, suggesting that SLC7A11 was a target of miR-27a-3p (**Figures 4G, H**).

### miR-27a-3p Regulates Erastin Induced Ferroptosis in NSCLC Cells

**Figure 5:**
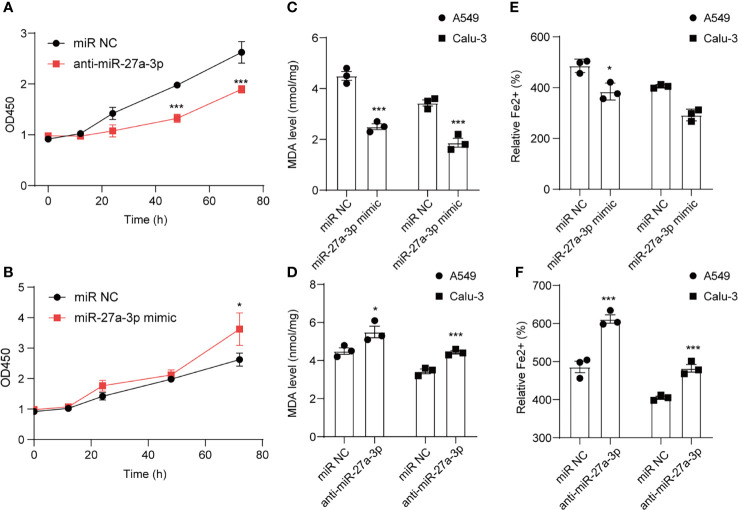
miR-27a-3p regulates erastin induced ferroptosis in NSCLC cells. The influence of miR-27a-3p on erastin induced ferroptosis on A549 and Calu-3 cells. Cell viability **(A, B)**, MDA level **(C, D)**, Fe^2+^ accumulation **(E, F)** were determined after miR-27a-3p mimic and anti-miR-27a-3p transfection. *P < 0.05 and ***P < 0.001.

**Figure 6:**
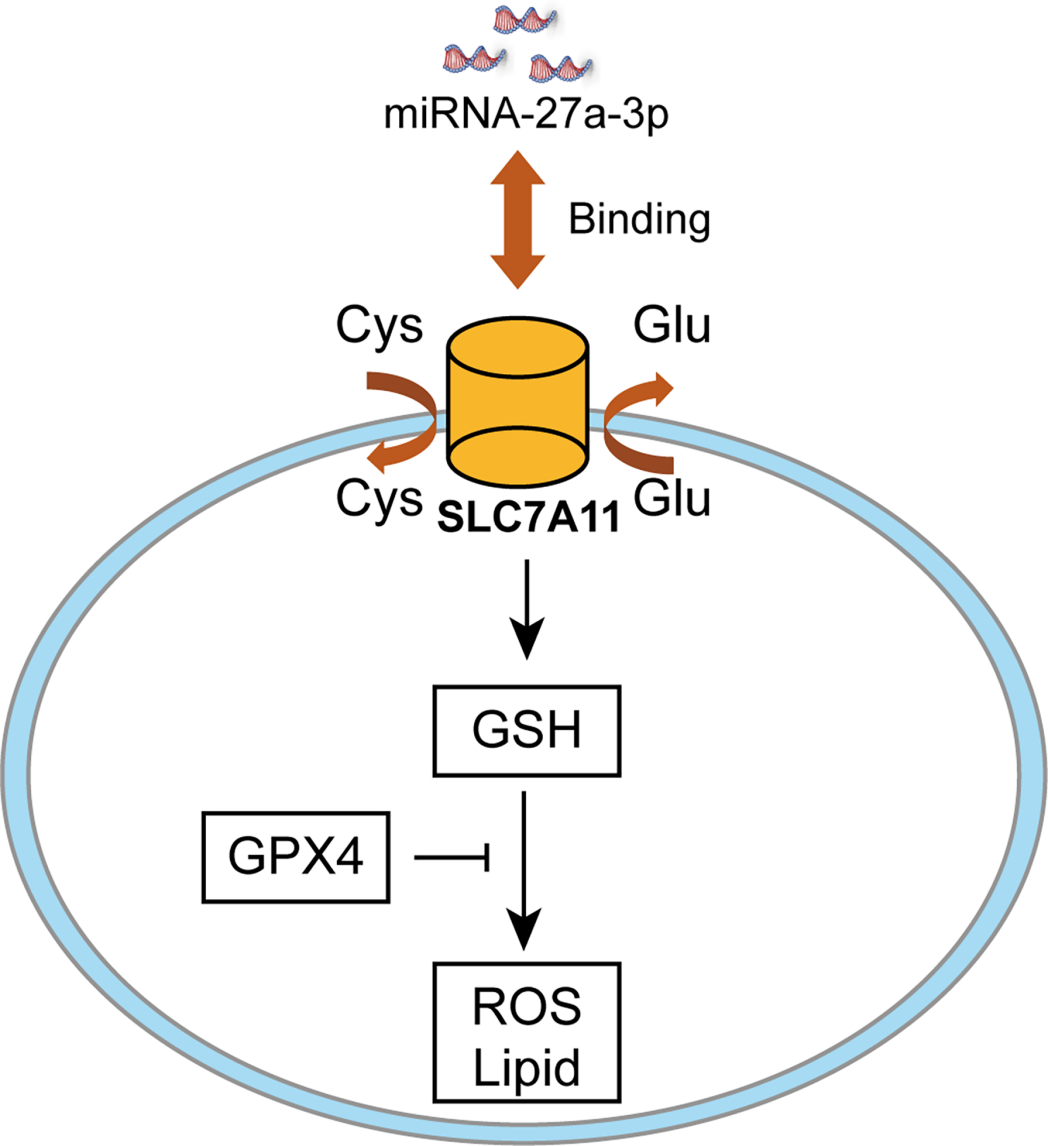
Schematic of the working details of miR-27a-3p/SLC7A11 in NSCLC.

CCK-8 cell viability assay was employed to analyze cell death after erastin treatment. Cell viability was reduced after downregulating miR-27a-3p in comparison with control (**Figure 5A**), suggesting that miR-27a-3p negatively modulated ferroptosis. To unearth miR-27a-3p’s role in ferroptosis, miR-27a-3p mimic was transfected into cells, and the data illustrated that upregulating miR-27a-3p contributed to the promotion of cell viability (**Figure 5B**). Similarly, we also assessed miR-27a-3p’s role in regulating MDA accumulation in NSCLC cells. The data indicated that the elevated MDA after erastin treatment was largely reversed upon overexpressing miR-27a-3p (**Figure 5C**). In addition, after erastin treatment, the overexpressed miR-27a-3p decreased Fe^2+^ levels in A549 and Calu-3 cells (**Figure 5E**). And the inhibition in endogenous miR-27a-3p caused by the antagonist led to the increase in MDA accumulation and intracellular Fe^2+^ levels in A549 and Calu-3 cells (**Figures 5D, F**). Of interest, miR-27a-3p exerted more significant effect on lipid peroxidation relative to iron accumulation (**p<0.01 *vs* *p<0.05), indicating that miR-27a-3p mediated the inhibition of ferroptosis majorly by regulating lipid peroxidation in NSCLC cells. Schematic of the working details of miR-27a-3p/SLC7A11 in NSCLC was shown in **Figure 6**.

## Discussion

NSCLC, the most common type of lung cancer, is further classified into adenocarcinoma (AC), squamous cell carcinoma (SCC), and large-cell carcinoma (LCC). As the pathogenesis process of NSCLC is very complex, the understanding of the progression of NSCLC is still limited. In recent years, extensive studies have revealed that miRNAs are important regulators in NSCLC progression. The abnormal expression of miRNAs can be used as a prognostic indicator for the treatment target of NSCLC.

Ferroptosis is a sort of programmed cell death, which is non-apoptotic and reported as regulated cell death in an iron and ROS-dependent form (13). The morphological characteristics of ferroptotic cells contained mitochondrial structure changes, along with nuclear noncontraction and plasma membrane rupture (18), usually generated by inhibited system xc- (i.e., erastin) (19). System xc- is the cystine/glutamate antiporter, which is responsible for importing extracellular cystine in exchange for intracellular glutamate (20). The cystine/glutamate antiporter xCT (SLC7A11) is a primary constituent of system xc- (21). The starvation of intracellular cystine brought about the exhaustion of the glutathione (GSH) level and consequent inactivation of GSH peroxidase 4 (GPX4) function (22, 23). The reduction of lipid hydroperoxides to lipid alcohols was executed by GPX4 (24). A high level of lipid ROS was triggered in the absence of GPX4 activity (25). Additionally, excessive iron also produced ferroptotic cell death by engendering ROS *via* the Fenton reaction (26, 27). As the key component of system xc-, SLC7A11 can mediate neutral amino acid uptake, such as Gln. The intracellular Gln pool is vital for continuous activation of mammalian target of rapamycin complex 1 (mTORC1) signaling, which mainly modulated cell growth, apoptosis, and autophagy. In erastin-triggered ferroptosis, the importation and metabolism of Gln brought in the generation of lipid ROS and the promotion of cell death. Accumulating evidence demonstrated that SLC7A11 played importantly in the progression and the survival of different carcinoma cell types, including breast, glioma, and lymphoma (28–30). Dixon et al. found that SLC7A11, a subunit that inhibits amino acid transporter, can accelerate erastin-induced cell death, while overexpression of SLC7A11 can inhibit erastin-induced cell death (19). Cystine uptake regulated by SLC7A11 is a rate-limiting step for biosynthesis of glutathione in organisms (31). Here we described the functional significance of SLC7A11 overexpression in NSCLC. SLC7A11 was overexpressed in the tissues of NSCLC, and its overexpression had a correlation with poorly prognostic status in NSCLC patients. Studies were showing that overexpressing SLC7A11 induced ferroptosis in NSCLC cells, and silencing SLC7A11 inhibited NSCLC cell proliferation and lipid peroxidation *in vitro*.

Pioneering works have found that SLC7A11 expression is regulated by various stimuli such as oxygen (32) and electrophilic agents (33). Later, emerging studies reveal that under various cellular stresses in a host of cancers, SLC7A11 is mostly adaptively upregulated to mitigate intracellular ROS and replenish GSH, thereby antagonizing cell death and resisting anticancer therapies. From the perspective of post-transcriptional modification, accumulating evidence demonstrates that SLC7A11 mRNA is derepressed by decreased miR-26b in human breast cancer and miR-375 in oral squamous cell carcinoma (34–36). And bioinformatic analysis predicted that SLC7A11 is targeted by different miRNAs, including has-mir-373 and has-mir-372 (37), miR-374b-5p and miR-26b-5p (38), and miRNA-126-3p/5p (39), but whether and how these miRNAs regulate SLC7A11 are not clear. In the present study, we predicted the candidate target genes for miR-27a-3p using TargetSan, miRanda, microT, PITA, miRmap, and PicTar platform. Our current data suggested that SLC7A11 was a potential target of miR-27a-3p. Further data revealed that rescued miR-27a-3p made NSCLC cells sensitive to erastin-induced ferroptosis, which greatly inhibited NSCLC cells ability *in vitro*. Collectively, miR-27a-3p, the firstly identified miRNA, mediated the cross-modulation amid apoptosis, autophagy, and ferroptosis. To sum up, we recapitulated that the reduced miR-27a-3p promoted ferroptosis mediated by SLC7A11, offering a split-new target for NSCLC’s diagnosis and treatment. There are some disadvantages in this study; the *in vivo* experiments are required to confirm the function of the miR-27a-3p in NSCLC. We will try our best to make up for the above deficiencies in our future work and research.

## Conclusions

We found that SLC7A11 was dramatically raised in NSCLC patients and cell lines, and SLC7A11 expression associated with prognosis in patients with NSCLC. Furthermore, our research shows that ferroptosis mediates the regulation of NSCLC development *via* targeting the miR-27a-3p/SLC7A11 pathway. These observations suggested that SLC7A11 is one target of miR-27a-3p, and the reduction of miR-27a-3p promoted ferroptosis mediated by SLC7A11 in NSCLC, offering a split-new direction for NSCLC’s diagnosis and treatment.

## Data Availability Statement

The original contributions presented in the study are included in the article/**Supplementary Material**. Further inquiries can be directed to the corresponding authors.

## Ethics Statement

Our research was approved by the institutional ethics committee of Huashan Hospital of Fudan University. The patients/participants provided their written informed consent to participate in this study.

## Author Contributions

SG and WD planned and designed the experiments and wrote the manuscript. XL performed cell biology experiments. XXL, NK, and MP acquired and analyzed metabolic experiments. MP provided pathological support. XL performed statistical analyses. All authors contributed to the article and approved the submitted version.

## Funding

The present work is supported by the study of the Special Professor of “Wanjiang Scholars” (2019).

## Conflict of Interest

The authors declare that the research was conducted in the absence of any commercial or financial relationships that could be construed as a potential conflict of interest.

## Publisher’s Note

All claims expressed in this article are solely those of the authors and do not necessarily represent those of their affiliated organizations, or those of the publisher, the editors and the reviewers. Any product that may be evaluated in this article, or claim that may be made by its manufacturer, is not guaranteed or endorsed by the publisher.

## Supplementary Material

Supplementary Figure 1 CCK8 assay detection of cell survival levels of Calu-3 induced by Erastin and Erastin plus ferrostatin-1 at different concentrations. **P < 0.05, **P < 0.01, and ***P < 0.001.*

Supplementary Figure 2 ROS expression levels of A549 cells treated with indicated compounds. **P < 0.05, **P < 0.01*, and ****P < 0.001.*

Supplementary Figure 3 SLC7A11 expression levels in SLC7A11 knockdown A549 and Calu-3 cells. sh-NC or sh-SLC7A11 (sh-SLC7A11 1# and sh-SLC7A11 2#) were separately transfected into A549 and Calu-3 cells and SLC7A11 expression levels were measured using qPCR **(A, B)**. **P < 0.05, **P < 0.01*, and ****P < 0.001.*

## Abbreviations

LC, lung cancer; NSCLC, non-small cell lung cancer; SLC7A11, Solute Carrier Family 7 Member 11; xCT, cystine/glutamate antiporter system xc-; GPX4, Glutathione Peroxidase 4; GSS, glutathione synthetase; miRNA, Micro-RNA; FBS, fetal bovine serum.
