# Supplementary figures and images for: MiR-27a-3p Promotes Non-Small Cell Lung Cancer Through SLC7A11-Mediated-Ferroptosis
Source: Front Oncol. 2021 Oct 13;11:759346. doi: 10.3389/fonc.2021.759346 (PMC8548660; doi:10.3389/fonc.2021.759346)

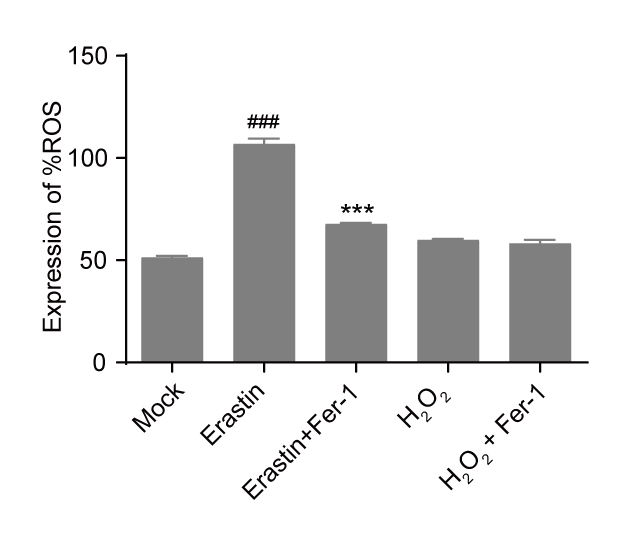

Supplement: Supplementary Figure 1 — CCK8 assay detection of cell survival levels of Calu-3 induced by Erastin and Erastin plus ferrostatin-1 at different concentrations. *P < 0.05, **P < 0.01, and ***P < 0.001. [file Image_1.tif]

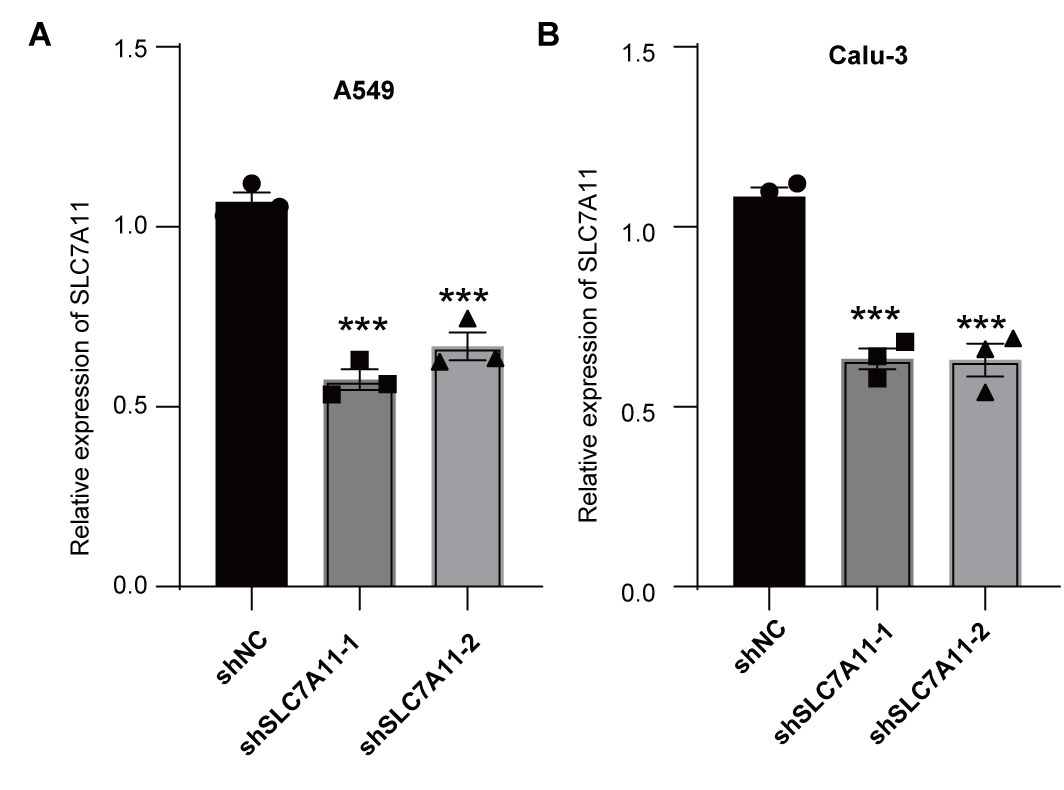

Supplement: Supplementary Figure 2 — ROS expression levels of A549 cells treated with indicated compounds. *P < 0.05, **P < 0.01, and ***P < 0.001. [file Image_2.tif]

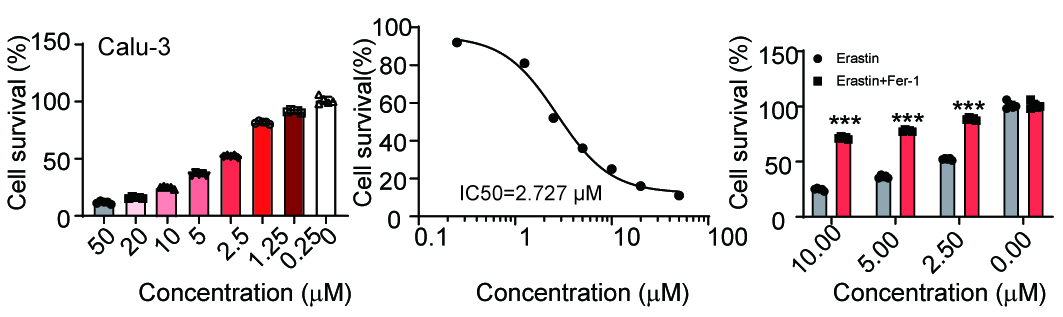

Supplement: Supplementary Figure 3 — SLC7A11 expression levels in SLC7A11 knockdown A549 and Calu-3 cells. sh-NC or sh-SLC7A11 (sh-SLC7A11 1# and sh-SLC7A11 2#) were separately transfected into A549 and Calu-3 cells and SLC7A11 expression levels were measured using qPCR (A, B). *P < 0.05, **P < 0.01, and ***P < 0.001. [file Image_3.tif]
