# Supplementary material for: MiR-27a-3p Promotes Non-Small Cell Lung Cancer Through SLC7A11-Mediated-Ferroptosis
Source: Front Oncol. 2021 Oct 13;11:759346. doi: 10.3389/fonc.2021.759346 (PMC8548660; doi:10.3389/fonc.2021.759346)
Supplement: Supplementary file 4 [file Table_1.docx]

**Supplementary Table 1 The primers for qRT-PCR**

| Gene | Forward primer sequence（5’-3’） | Reverse primer sequence（5’-3’） |
| --- | --- | --- |
| Human SLC7A11 | TGCTGGGCTGATTTTATCTTCG | GAAAGGGCAACCATGAAGAGG |
| Human GAPDH | ATCATCCCTGCATCCACT | ATCCACGACGGACACATT |
| miR-27a-3p | ATGGTTCGTGGGTTCACA | GTGGCTAAGTTCCGACG |
| U6 | ATACAGAGAAGATTAGCATGGCCCCTG | ACACGCAAATTCGTGAAGCGTTCCATATTT |
